# Nosocomial Transmission of SARS-CoV-2 Involving Vaccinated Health Care Workers

**DOI:** 10.1128/spectrum.01532-21

**Published:** 2022-01-05

**Authors:** Laura Pérez-Lago, Marina Machado, María de Mar Gómez-Ruiz, Pedro J. Sola-Campoy, Sergio Buenestado-Serrano, Victor Manuel de la Cueva-García, Marta Herranz, Cristina Andrés Zayas, Ignacio Sánchez-Arcilla, Rubén Francisco Flores-García, Nieves López-Fresneña, Sonia García de San José, Pilar Catalán, Patricia Muñoz, Darío García de Viedma

**Affiliations:** a Servicio de Microbiología Clínica y Enfermedades Infecciosas, Gregorio Marañón General University Hospital, Madrid, Spain; b Instituto de Investigación Sanitaria Gregorio Marañón (IiSGM), Madrid, Spain; c Genomics Unit, Gregorio Marañón General University Hospital, Madrid, Spain; d Servicio de Prevención de Riesgos laborales, Gregorio Marañón General University Hospital, Madrid, Spain; e Gerencia, Gregorio Marañón General University Hospital, Madrid, Spain; f Servicio de Medicina Preventiva y Gestión de Calidad, Gregorio Marañón General University Hospital, Madrid, Spain; g CIBER Enfermedades Respiratorias (CIBERES), Madrid, Spain; h Departamento de Medicina, Universidad Complutense, Madrid, Spain; Houston Methodist Hospital

**Keywords:** COVID-19, SARS-CoV-2, transmission, vaccination, nosocomial, genomics

## Abstract

COVID-19 vaccination has proven to be effective at preventing symptomatic disease but there are scarce data to fully understand whether vaccinated individuals can still behave as SARS-CoV-2 transmission vectors. Based on viral genome sequencing and detailed epidemiological interviews, we report a nosocomial transmission event involving two vaccinated health care-workers (HCWs) and four patients, one of them with fatal outcome. Strict transmission control measures, as during the prevaccination period, must be kept between HCWs and HCWs-patients in nosocomial settings.

**IMPORTANCE** COVID-19 vaccination has proven to be effective at preventing symptomatic disease. Although some transmission events involving vaccinated cases have also been reported, scarce information is still available to fully understand whether vaccinated individuals may still behave as vectors in SARS-CoV-2 transmission events. Here, we report a SARS-CoV-2 nosocomial transmission event, supported on whole genome sequencing, in early March 2021 involving two vaccinated HCWs and four patients in our institution. Strict transmission control measures between HCWs and HCWs - patients in nosocomial settings must not be relaxed, and should be kept as strictly as during the prevaccination period.

## OBSERVATION

COVID-19 vaccination has proven to be effective at preventing symptomatic disease (1) and reducing transmission from vaccinated infected individuals to other household members (2). Although some transmission events involving vaccinated cases have also been reported (3, 4), scarce information is still available to fully understand whether vaccinated individuals may still behave as vectors in SARS-CoV-2 transmission events (5). Here, we report a SARS-CoV-2 nosocomial transmission event, supported on whole genome sequencing, in early March 2021 involving two vaccinated health care-workers (HCWs) and four patients in our institution (Madrid, Spain). The findings were the result of a program running in our institution in which all SARS-CoV-2 reverse transcriptase-PCR (RT-PCR; TaqPath COVID-19 CE- 97 IVD RT-PCR kit; Thermo Fisher Scientific, USA) positive specimens from HCWs and those from patients due to nosocomial acquisitions are systematically sequenced. The study was approved by the ethical research committee of Gregorio Marañón Hospital (REF: MICRO.HGUGM.2020-042). Informed consent was obtained from the patients for publication of this report.

Whole genome amplification was done with an Artic_nCov-2019_V3 panel of primers (Integrated DNA Technologies, Inc., Coralville, IA) (artic.network/ncov-2019). Libraries were prepared using the Nextera DNA Flex Library Preparation Kit (Illumina lnc, CA) and sequenced in pools on the Miseq system (Illumina Inc, CA). An in-house analysis pipeline was applied on the sequencing reads (https://github.com/MG-IiSGM/covid_multianalysis). Briefly, the pipeline involves (i) removal of human reads with Kraken; (ii) pre-processing and quality assessment of fastq files using fastp v0.20.1 (arguments: –cut tail, –cut-window-size, –cut-mean-quality, -max_len1, -max_len2) and fastQC v0.11.9; (iii) mapping with BWA v0.7.17 and variant calling using IVAR v1.2.3, using the Wuhan-1 sequence (NC_045512.2) as reference and (iv) calibration of occasional low coverage positions using joint variant calling. Data supporting the findings of this study (FastA files) are openly available in GISAID at https://www.gisaid.org/. Reference numbers (EPI_ISL_2235521, EPI_ISL_2235522, EPI_ISL_2235523, EPI_ISL_2235524, EPI_ISL_2235525, EPI_ISL_2235526).

**FIG 1:**
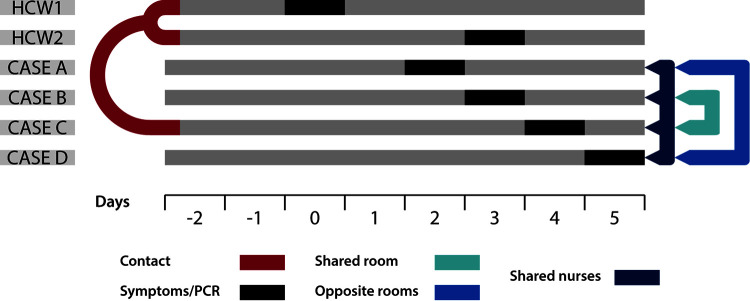
Chronology of events and epidemiologically relevant features (cases sharing a room or staying in opposite rooms, and cases sharing nurses). Contact days and symptoms onset/PCR are indicated (PCRs were performed the same day of symptoms onset).

Two COVID-19 vaccinated professionals (HCW1 and HCW2) shared an identical strain (variant B.1.1.7; zero single nucleotide polymorphisms [SNPs]) and developed mild symptoms on Days 0 and 3 (Fig. 1). The same strain was identified in four patients (cases A–D; zero SNPs between Case A-C sequences and 1 SNP with Case D), who had stayed in the same ward and were diagnosed in consecutive days (Days 2–5, in all of them the SARS-CoV-2 RT-PCR was positive at their symptoms onset; Fig. 1).

The epidemiological investigation, triggered by WGS results, to clarify the nature of the outbreak, identified different factors that suggest different transmission links within the cluster (Fig. 1). Firstly, HCW1 and HCW2 worked together and had had close contact during a morning social meeting on Day −2. Secondly, HCW1 and HCW2 had attended Case C on Day −2, 6 days before positive COVID-19 diagnosis of Case C. In addition, Cases A–D had shared the same nursing team throughout all their hospital stay, Cases B and C shared a room; Cases A and D stayed in two opposite rooms in the same corridor were doors had been kept open; Case D was a 85-year-old patient, with cognitive impairment (Alzheimer’s disease) and inability to walk due to a right pertrochanteric fracture; highly dependent and needing help for walk, transferring, wash, and dress (Barthel scale of 35 points). All these factors could explain the subsequent transmission of the strain from Case C to the other cases (A, B and D) within the ward.

**FIG 2:**
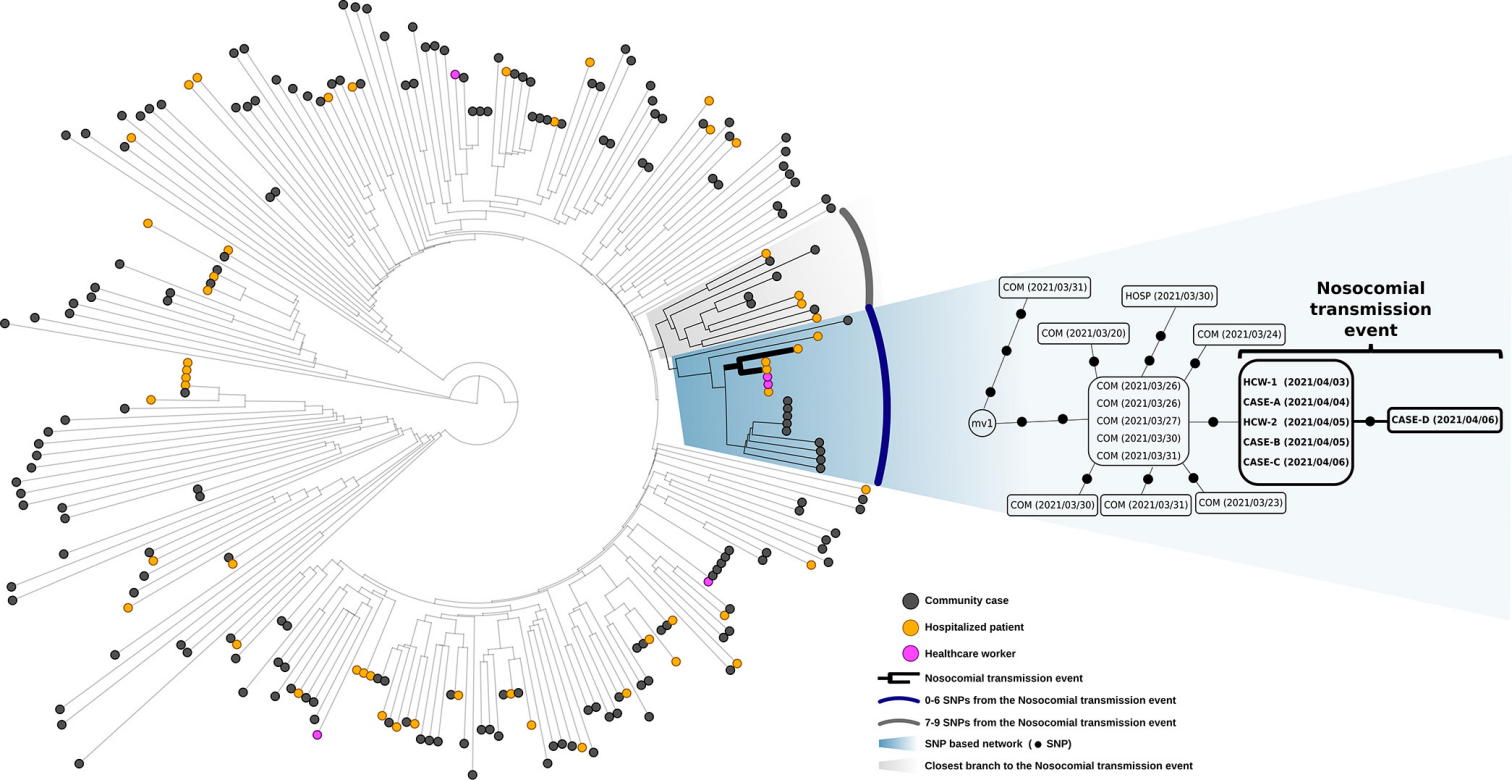
Left side: phylogenetic tree obtained from the analysis of the SARS-CoV-2 sequences from community and hospitalized cases diagnosed along the 2 weeks before the diagnosis of HCW-1, the first case in the event in study. Right side: network of genomic relationship for the cases involved in the nosocomial transmission event in study and those included in the closest neighboring branch are zoomed out. In the network of relationships, the cases within a box share identical sequences between them and each dot corresponds to 1 SNP. COM, community case; HOSP, hospitalized case; HCW, healthcare worker. Diagnosis dates as per SARS-CoV-2 RT-PCR are indicated within brackets. mv1, median vector corresponding to the node in the network not occupied by the sequences available.

To evaluate the potential involvement of other COVID-19 cases, both within or outside the hospital, in the nosocomial transmission event we performed a wider genomic integrate analysis. We compared (Fig. 2) the SARS-CoV-2 sequences from the six cases in study with the sequences from the 45 hospitalized cases, three HCWs and 189 community cases diagnosed along the 2 weeks preceding the diagnosis of the first case in our event (HCW1). This sample was obtained as part of our systematic sequencing of a random sample of 50–75 sequencing cases among those diagnosed each week. No other hospitalized or community patient, nor HCW, shared (0 SNPs) the SARS-CoV-2 strain involved in the nosocomial transmission event in study. All but one of the sequences from the 48 hospitalized cases/HCWs differed in >7 SNPs from the strain in study (Fig. 2). Only one sequence showed a shorter distance (three SNPs), but it was still unrelated to the outbreak strain as it lacked one SNP present in all the cases involved in the transmission event in study and, in addition, it harbored two private SNPs not shared by any other of the cases (Fig. 2). Five cases from the community, diagnosed within the week preceding HCW-1 diagnosis, shared a strain whose sequence preceded in one SNP to the set of SNPs shared by the outbreak strain (Fig. 2). These findings (i) reinforce the HCW-HCW transmission as the most likely explanation to the fact that both HCWs shared an identical SARS-CoV-2 strain; (ii) strongly minimize the direct involvement of other additional cases, either within or outside the hospital, in the transmission event in study; and (iii) suggest a likely community origin for the first HCW infected.

HCW1 and HCW2 had received their second vaccine dose (BNT161b2) 1 month before their COVID-19 diagnosis and developed immunological responses leading to anti-SARS-CoV2 IgGs levels of 8737 and 8801 UA/ml, respectively (SARS-CoV-2 IgG II Quant reagent kit, Abbott, Chicago, IL). Their serological response was measured within our program of systematically analyze the postvaccination serological status in all HCWs. Both HCWs experienced symptomatic COVID (mild symptoms) and the low RT-PCR Ct values obtained from their specimens (Ct values 18 and 17) allowed us to infer high SARS-CoV-2 titers. The two HCWs could be diagnosed due to their mild symptoms, however asymptomatic vaccinated HCWs might have a role in cryptic transmissions (6). Systemic vaccines are expected to offer only limited protection against viral replication within the upper airways (7), which might explain the high viral titers in nasopharyngeal specimens in the vaccinated HCWs in our study.

The 1-day contact between HCW1, HCW2, and Case C on Day −2 was sufficient to lead to a transmission event involving all three individuals. Subsequent transmission within Case C´s ward led to a nosocomial outbreak involving another three cases, one with a fatal outcome. Strict transmission control measures between HCWs and HCWs—patients in nosocomial settings must not be relaxed, and should be kept as strictly as during the prevaccination period.

## References

[1] Egunsola OM, Dowsett LE, Clement FM, on behalf of the University of Calgary Health Technology Assessment Unit. 2021. Transmissibility of COVID-19 among Vaccinated Individuals: Targeted Literature Search.

[2] Shah AS, Gribben C, Bishop J, et al. 2021. Effect of vaccination on transmission of COVID-19: an observational study in healthcare workers and their households. medRxiv. doi:10.1101/2021.03.11.21253275.

[3] Cavanaugh AM, Fortier S, Lewis P, Arora V, Johnson M, George K, Tobias J, Lunn S, Miller T, Thoroughman D, Spicer KB. 2021. COVID-19 outbreak associated with a SARS-CoV-2 R.1 lineage variant in a skilled nursing facility after vaccination program—Kentucky, March 2021. MMWR Morb Mortal Wkly Rep 70:639–643. doi:10.15585/mmwr.mm7017e2. 33914720 PMC8084128

[4] Donadio C, Rainone A, Gouronnec A, Belmin J, Lafuente-Lafuente C. 2021. Asymptomatic COVID-19 cases among older patients despite BNT162b2 vaccination: A case series in a geriatric rehabilitation ward during an outbreak. The J Infection 83:119–145. doi:10.1016/j.jinf.2021.04.004. PMC8038862 33852930

[5] ECDC. 2021. Risk of SARS-CoV-2 transmission from newly-infected individuals with documented previous infection or vaccination. Stockholm, Sweden: ECDC.

[6] Schiavone M, Gasperetti A, Mitacchione G, Viecca M, Forleo GB. 2021. Response to: COVID-19 re-infection. Vaccinated individuals as a potential source of transmission. Eur J Clin Invest 51:e13544. doi:10.1111/eci.13544. 33725359 PMC8250045

[7] Bleier BS, Ramanathan M, Jr, Lane AP. 2021. COVID-19 vaccines may not prevent nasal SARS-CoV-2 infection and asymptomatic transmission. Otolaryngol Head Neck Surg 164:305–307. doi:10.1177/0194599820982633. 33320052

